# Content-based analysis of Ki-67 stained meningioma specimens for automatic hot-spot selection

**DOI:** 10.1186/s13000-016-0546-7

**Published:** 2016-10-07

**Authors:** Zaneta Swiderska-Chadaj, Tomasz Markiewicz, Bartlomiej Grala, Malgorzata Lorent

**Affiliations:** 1 Warsaw University of Technology, 1 Politechniki Sq., 00-661 Warsaw, Poland; 2 Military Institute of Medicine, 128 Szaserow St, 04-141 Warsaw, Poland

**Keywords:** Whole slide images, Hot-spot, Meningiomas, Image analysis

## Abstract

**Background:**

Hot-spot based examination of immunohistochemically stained histological specimens is one of the most important procedures in pathomorphological practice. The development of image acquisition equipment and computational units allows for the automation of this process. Moreover, a lot of possible technical problems occur in everyday histological material, which increases the complexity of the problem. Thus, a full context-based analysis of histological specimens is also needed in the quantification of immunohistochemically stained specimens. One of the most important reactions is the Ki-67 proliferation marker in meningiomas, the most frequent intracranial tumour. The aim of our study is to propose a context-based analysis of Ki-67 stained specimens of meningiomas for automatic selection of hot-spots.

**Methods:**

The proposed solution is based on textural analysis, mathematical morphology, feature ranking and classification, as well as on the proposed hot-spot gradual extinction algorithm to allow for the proper detection of a set of hot-spot fields. The designed whole slide image processing scheme eliminates such artifacts as hemorrhages, folds or stained vessels from the region of interest. To validate automatic results, a set of 104 meningioma specimens were selected and twenty hot-spots inside them were identified independently by two experts. The Spearman rho correlation coefficient was used to compare the results which were also analyzed with the help of a Bland-Altman plot.

**Results:**

The results show that most of the cases (84) were automatically examined properly with two fields of view with a technical problem at the very most. Next, 13 had three such fields, and only seven specimens did not meet the requirement for the automatic examination. Generally, the Automatic System identifies hot-spot areas, especially their maximum points, better. Analysis of the results confirms the very high concordance between an automatic Ki-67 examination and the expert’s results, with a Spearman rho higher than 0.95.

**Conclusion:**

The proposed hot-spot selection algorithm with an extended context-based analysis of whole slide images and hot-spot gradual extinction algorithm provides an efficient tool for simulation of a manual examination. The presented results have confirmed that the automatic examination of Ki-67 in meningiomas could be introduced in the near future.

**Electronic supplementary material:**

The online version of this article (doi:10.1186/s13000-016-0546-7) contains supplementary material, which is available to authorized users.

## Background

The quantitative examination of histological tissues subject to immunostain tests is a basic method of recognizing a tumour, choosing optimal therapy and defining the prognostic indicators. One of the most important markers is the proliferation marker Ki-67/MIB-1. The value of this marker reflects the rate of tumour cell proliferation, and indicates the speed of tumour growth, as well as the degree of malignancy. In this study, we focus on central nervous system tumours. Meningiomas, which are the most frequent primary intracranial tumour, can be differentiated by the proliferation index into meningothelial (WHO I), atypical (WHO II), and anaplastic (WHO III). The index can also provide prognostic factors, as well as correlate with tumour recurrences [1, 2].

The methodology of the quantitative evaluation of tumour proliferation marked via Ki-67 stain is still under debate and there is no full agreement on any single strategy. This problem, with a lot of possible influences on resultant indicators, was discussed in [2]. However, due to the most frequent assumptions in the WHO classification, as well as restrictions in manual (greater) and automatic (lesser) examination, we should select a set of high power fields of view (FOV). These fields are called hot-spots and they serve as a base to calculate the Ki-67 index.

In routine diagnostic practice, representative hot-spot areas are manually selected by histopathologists using visual examination of Ki-67 immunostained specimens at a low magnification (both, in microscope or virtual slide). This process might lack reproducibility and affect the Ki-67 due to the subjectivity of evaluation [3, 4]. First of all, the histological criteria of hot-spot selection are flexible. The selected fields should represent areas of high Ki-67 index, but also different tumour localizations. Also, in tumours of the central nervous system, the bordering regions are suggested where the high power fields indicated in the WHO recommendation reflect high concentrations of tumour cells [1]. So, this ambiguity complicates the selection of the proposed procedure. Finally, the influence of many factors, such as the expert’s experience, fatigue, previously viewed preparations and external factors, is also significant.

Recent developments in microscopic glass scanners, as well as easily accessible computational machines (computing servers, clouds, power computers with advanced graphical units) have led to the increased potential of digital pathology. The goal has changed from single image processing to whole slide image (WSI) analysis. While a lot of algorithms for cell segmentation and counting in the images are described in the literature [5, 6], the approaches to hot-spot finding in whole slide images are still under design. Most of these address the analysis of tissues treated with a standard Hematoxylin & Eosin stain [7, 8]. Recently, an algorithm for microvessel analysis has been described [9] and [10], where the authors present a method for increasing the visibility of the positive nuclei on low resolution images. However, hot-spot fields are still selected manually. In another recent paper [11], the automated selection of hot-spot algorithms was proposed. The adaptive step finding technique has been applied to increasing the computational efficiency and performance of hot-spot detection. Despite this significant progress, the problems of artifacts, specimen quality, and spatial distribution of the selected hot-spot fields were not discussed in this paper. Indeed, one of the significant problems in whole slide examination is the occurrence of areas of hemorrhages (vs. erythrocytes both intravascular or extravasated), and vessel walls, which are also stained with brown. Therefore, there is a need to differentiate these from the areas of tumour proliferation. Next, the folds are present in a lot of specimens, even the most carefully prepared. They are the natural effect of specimen cutting (formerly curling and straightening of a tissue section). Their presence can also interfere with the automatic examination of WSI.

Despite the question of quality mentioned above, the problem of the spatial distribution of the selected hot-spot fields is also very compound. Some solutions to the specific tasks of hot-spot selections were presented in our previous studies [12–14]. In this paper, we develop them with the algorithm of specimen fold detection, vessel elimination, small artifact caused error prevention, and the WSI processing strategy to offer a complete system for automatic hot-spot selection in Ki-67 stained meningiomas specimens. The solution is based on mathematical morphology, texture analysis (Unser and Local Binary Patterns approaches), different color representations and penalty functions. A comparison of automatic results with experts’ Ki-67 examinations is also included and discussed.

## Methods

The 104 analyzed cases come from the archives of the Department of Pathomorphology from the Military Institute of Medicine in Warsaw, Poland. The cases come from the last 5 years. The data collection was approved by the IRB of the Military Institute of Medicine. The Ki-67/MIB-1 immunohistochemical stained procedure was performed using Dako Autostainer Link and the following chemicals: FLEX Monoclonal Mouse Anti-Human Ki-67 Antigen Clone MIB-1 Ready-to-Use (Link) ref no. IR626 from Dako. The staining was visualized using EnVision™ FLEX Target Retrieval Solution from Dako, according to the procedure described in the user manual. Most of the selected cases had Ki-67 staining performed on the day of examination, and only a few of them have recently completed markers. A 3DHISTECH Pannoramic 250 Flash II scanner was used in the WSI acquisition process. The whole slide images were acquired under a 20 × lens with an effective resolution of 0.38895 μm per pixel. Digital images were reviewed using Matlab software and a dedicated OpenSlide library [15] to read WSI files. To ensure comparability of the area examined by the expert in the microscope as one field of view and the area of quantification chosen from digital WSI, the size of the rectangle which covered the same area as the microscopic circular FOV was determined. On the assumption that the microscopic field of view at 400× magnification represents around 0.12 mm^2^ of a tissue, the size of the digitized FOV was 1024 × 766 pixels.

### General WSI processing scheme

**Fig. 1:**
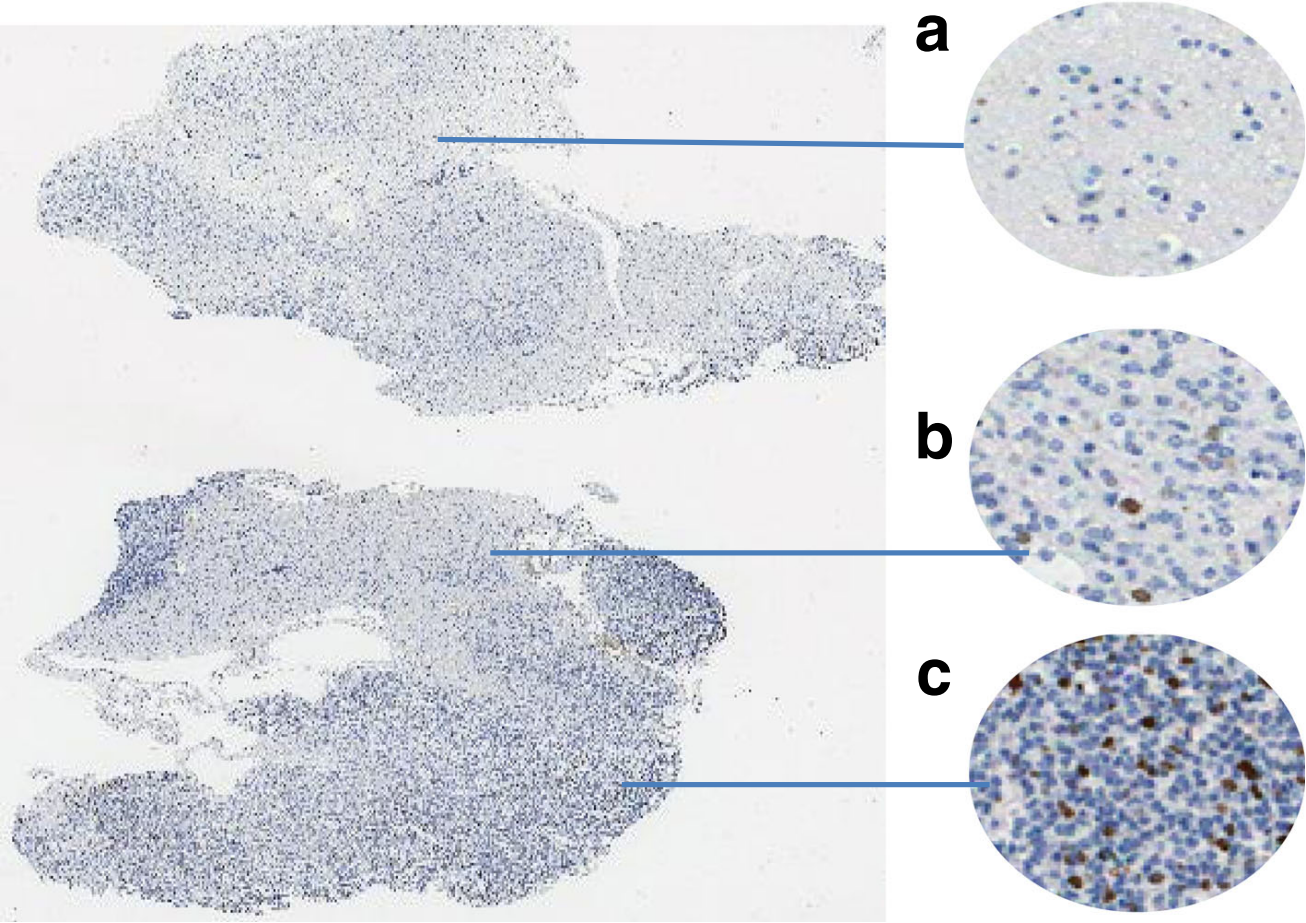
An example of WSI with FOVs (**a**,**b**,**c**)

The automatic hot-spot detection in the WSI requires a number of processing steps before the actual analysis of spatial immunopositive cell concentration. We can enlist such tasks as the creation of a specimen map, detection of hemorrhage/erythrocyte areas and folds of tissue, if present, and segmentation of immunopositive cells to build a map for their spatial distribution analysis. In our opinion, the original full resolution is not the best solution to perform such processing. So, first, the reduced resolution images directly available in *mrxs* format were studied to select the most appropriate one from the point of view of the accuracy of the result and low computational time. As mentioned in paper [14], the most useful is the eight-fold reduction of WSI resolution that still preserves the possibility of the recognition of specific cells. Thus, hot-spot detection is effectively realized on images of a size of about 17 000 × 7 000 pixels and depends on the settings of the scanning area in scanner software. Examples of WSI and FOVs are presented in Fig. 1, where A and B present FOVs with low levels of Ki-67 reaction, and C presents an example of a hot-spot area.

**Fig. 2:**
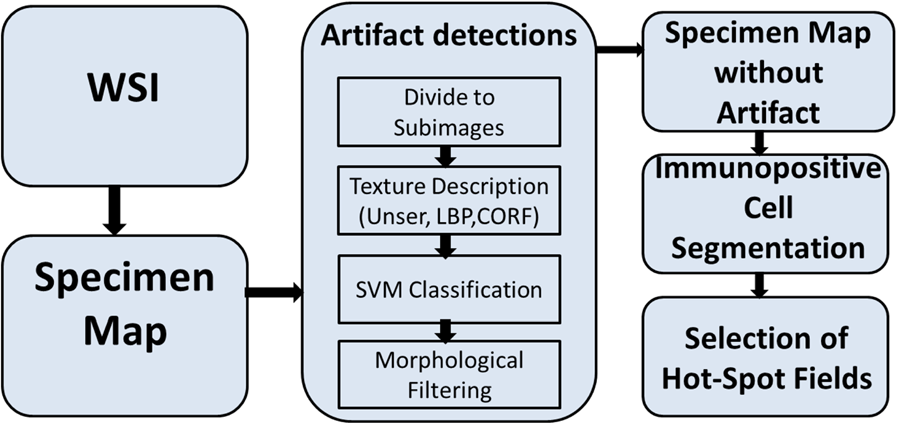
The general diagram of the hot-spot algorithm

The sequential processing steps of automatic hot-spot detection are ordered in the flow diagram presented in Fig. 2. While some of them must be processed in serial manner, others can be realized in parallel processing. This technique allows for a significant reduction of processing time, but the final implementation depends on the computing machine (number of cores and type of GPU). The specific processing steps with this method are described below.

Only a few steps of the proposed algorithm were presented in the preliminary papers [12–14, 16]. They were verified on an initial limited data set (10–15 cases) and their parameters were adjusted. There were focused on hemorrhage [12] and specimen fold [16] detections based on the textural features. Now, we extend our artifact detection machine for a vessel wall elimination procedure. Moreover, color artifact elimination was designed and added to prevent false immunopositive cell extraction. All these steps are proposed based on an extensive analysis of a hue dataset to discover as many artifact problems as possible that could occur in WSI processing.

### Creation of the specimen map

In the first step, a map of the specimen is created. The output WSI obtained from the scanner covers the scanned area in different colors representing a tissue and white outlined margin. The non-scanned areas are represented in black color (zero values in RGB representation respective to three color channels). Thus, all black pixels of WSI are recognized as a non-tissue region. The remaining region is thresholded and morphologically filtered [17]. The map of the specimen is based on the differencing image of B and R color components from the RGB color space, which was processed with the Otsu thresholding method [18]. Thereafter, morphological filtration is applied. This includes operations such as dilation and erosion with a small structural element (a disk 5–8 pixels in size), and removal of small holes and structures via an *imfill* operation to obtain a coherent map of the tissue.

### Texture description and classification

The detection of hemorrhage areas (vs. erythrocytes both intravascular or extravasated) and folds is the most crucial step to obtain a high level of accuracy from quantitative examination of specimens. Although we do not face these problems in all specimens, if they occur, the detected hot-spot fields can represent hemorrhage/erythrocytes instead of proliferation cells. This is because in some cases blood cells react with the antigen and are stained in brown as immunopositive tumour cells. Thus, their elimination from the region of interest is necessary. Also, a tissue fold disfigures real cell distribution which may result in local overestimation of immunopositive cell concentration. To detect such unwanted regions, local texture descriptions are created. Two methods were used: Unser features [19] and Local Binary Patterns (LBP) [20–22].

The Unser textures is a method which is based on the normalized probability applied to the pixel intensity of the image. The modified formulas of Unser features are based on the histograms of the sum and difference of images [19], which are counted locally in each pixel on examination of its neighborhood region Ω [12, 14]. In such a manner, the following features are counted: mean, variance, homogeneity, contrast, energy, correlation, cluster shade and cluster prominence. In this textural description, the parameters of resolution and radius influence the characterization of the local structures in specimens. In the case of a radius which is too small, the impact of surroundings is slight, and as a result the heterogeneity is strong.

**Fig. 3:**
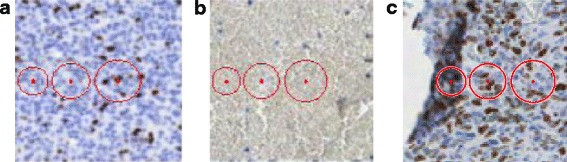
Example Ω regions with the radius of 8, 10, and 12 pixels imposed on three types of specimen structures: **a** tumour, **b** blood, and **c** fold

An illustration of this problem is included in Fig. 3 where examples of Ω regions with the radius of 8, 10 and 12 pixels are imposed on three types of specimen structures: tumour, blood, and fold. The optimal radius of regions for the best representation of these textures should be within this range. Please be aware that the resolution should be so selected, so the objects (such as the nuclei of tumour cells) are represented by at least a few pixels.

**Fig. 4:**
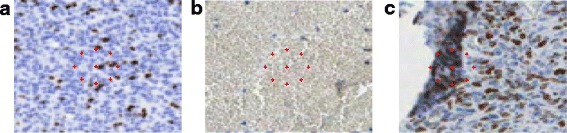
An example distribution of sampling points on the same specimen structures as in Fig. 3: **a** tumour, **b** blood, and **c** fold

The second type of texture description was the Local Binary Patterns method. The LBP method, presented in [20–22], has recently gained a wide range of different applications in image processing [23–25]. This method is based on the assumption that texture has two complementary aspects, a pattern and its strength. In [21] it is presented in the more generic, revised form of the LBP operator, without limitations to the size of the neighborhood and to the number of sampling points. We have a few different forms of LBP available, such as: uniform LBP, rotation-invariant LBP or uniform rotation-invariant LBP. The advantage of LBP is its efficiency of analyzing textures. The LBP method has a simple theory and combines properties of structural and statistical texture analysis methods. Paper [26] showed that it is preferable to use rotational invariant features for the analysis of the given biological structures, mostly anisotropic. In this method, a set of points around the central pixel distributed with a selected radius distance is also taken into account. The number of points affects method sensitivity and specificity to recognize a compact structure and has a significant impact on computational time. Figure 4 presents an example of the distribution of sampling points with a radius of 12 pixels over the same specimen structures as in Fig. 3.

The crucial problem for a useful textural description is the choice of color components. The color components used should allow differentiation between regions such as tumour, hemorrhage/erythrocytes and fold irrespective of the percentage of immunopositive cells. In previous studies [12, 16] we observed the significant impact of RGB components on the variability in values of texture characteristics. In paper [12] we proposed a method to image color representation, independent of the percentage of immunopositive cells. This method is based on introducing an additional color representation in the form of a sum of u (from CIE Luv color space) multiplied by 512 and C (CMYK color space) components. In order to describe the analyzed textures, the texture features were determined for each analyzed color component.

Not all features are useful in the classification process. The Fisher’s linear discriminant [27] was applied to assess the suitability of individual features. For data classification (tumour area, hemorrhage/erythrocytes, and fold), the Support Vector Machine (SVM) with Gaussian kernel function [28] was applied [12, 16]. It should be noted that one SVM is able to separate data into only two labeled classes.

Hence, three classes were taken into account: firstly, one SVM classifier was applied to differentiate between the tumour and hemorrhage/erythrocytes, and later elimination of the unwanted structures, the second SVM classifier was applied for possible fold detection in the tumour area.

The important aspect is good correlation between a tissue, the texture features and class. So, very important is how learning data were prepared. The data lying in margin regions can have an adverse influence on the class separation of the hyperplane of SVM. This problem is observed especially in the differentiation between hemorrhage and tumour. Indeed, the hemorrhage frequently penetrates the tumour region, and the border between these two is ambiguous. To present data for specific class clearly a slightly larger radius (higher data integrity within classes) is used in the learning process than in testing mode. In such a way, the SVM classifiers obtained a greater ability to adapt to the non-linear border between the regions.

### Vessel wall detection

**Fig. 5:**
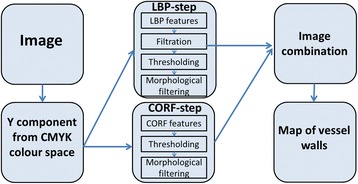
Diagram of vessel wall detection algorithm

In some specimens, an immunopositive reaction is also observed in the vessel walls. Although this should be recognized as an artifact, the repetition of acquiring such specimens in most cases cannot eliminate the problem. Thus, the examination must be performed with this artifact taken into account. The essence of the problem lies in the fact that vessel walls stained with brown can be classified as immunopositive cells. Thus, they should be eliminated from the image before the immunopositive cell segmentation step. In order to detect and eliminate them from the image, we propose a solution based on the LBP texture description (see above for details) and the Combination of Receptive Fields (CORF) method [29, 30]. The CORF model is inspired by the biological role of simple cells. This method can be used for contour detection, based as it is on contrast changes [29–31]. Combination of these two methods allows for the detection of vessel walls of different shapes and color intensity. Figure 5 presents a schema of the algorithm for vessel detection.

**Fig. 6:**
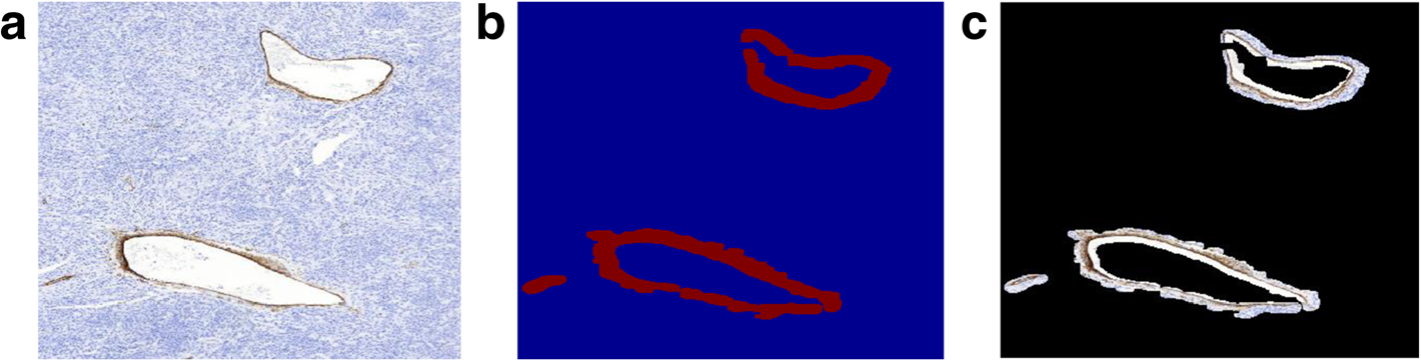
An example of vessel walls on an image (**a**), a map of detected vessel walls (**b**), and detected vessel walls (**c**)

The proposed processing was performed for the Y component from the CMYK color space. This color component allows for the best differentiation of the vessel walls in the image. In the first step, we detected vessels based on the LBP texture analysis method. In order to achieve a coherent wall image, we applied mean filtration, thresholding, and mathematical morphology operations. In the second step, we detected vessels based on the CORF method combined with thresholding and mathematical morphology operations. The mathematical morphology operations allow for the elimination of small structures from the final image. Images obtained in previous steps were combined in one map, which includes detected vessels. As a result, we achieved an image map with vessels. Based on this, we were able to eliminate this area from the processed WSI area. Examples of vessels walls in the image, and a detected vessel wall map are presented in Fig. 6.

### Immunopositive cell segmentation

The last step before the analysis of the local cell concentration to hot-spot detection was an immunopositive cell segmentation. For this step, only those regions previously recognized as tumours without hemorrhages and folds were considered. To recognize immunopositive cells different approaches have been proposed in the literature [5, 6, 32]. However, in our case the reduced resolution of the image had such an effect that the specific cell nuclei were represented by only a few pixels (e.g. four or six pixels) with significant blurring. Thus, a complex analysis, such as area, texture, shape, etc., was impossible. The simple method for cell segmentation is thresholding of the most differentiating color channel. However, the difference between the intensity of immunoreaction around the whole slide and between the different WSI made it difficult to choose the proper threshold value.

The above restrictions led to the application of an extended regional extreme [17] approach (from a family of mathematical morphology methods) to recognize the immunopositive cells. This transformation is defined as recognizing extreme regions where the values are higher (maxima) or lower (minima) then the surrounding areas by a selected *h* value. So, each regional extreme (point or set of points with the same value) is adapted for the surrounding region whose value equals the value of the extreme reduced by *h*. In this manner, local property regions are utilized, but without the control of the object areas.

**Fig. 7:**
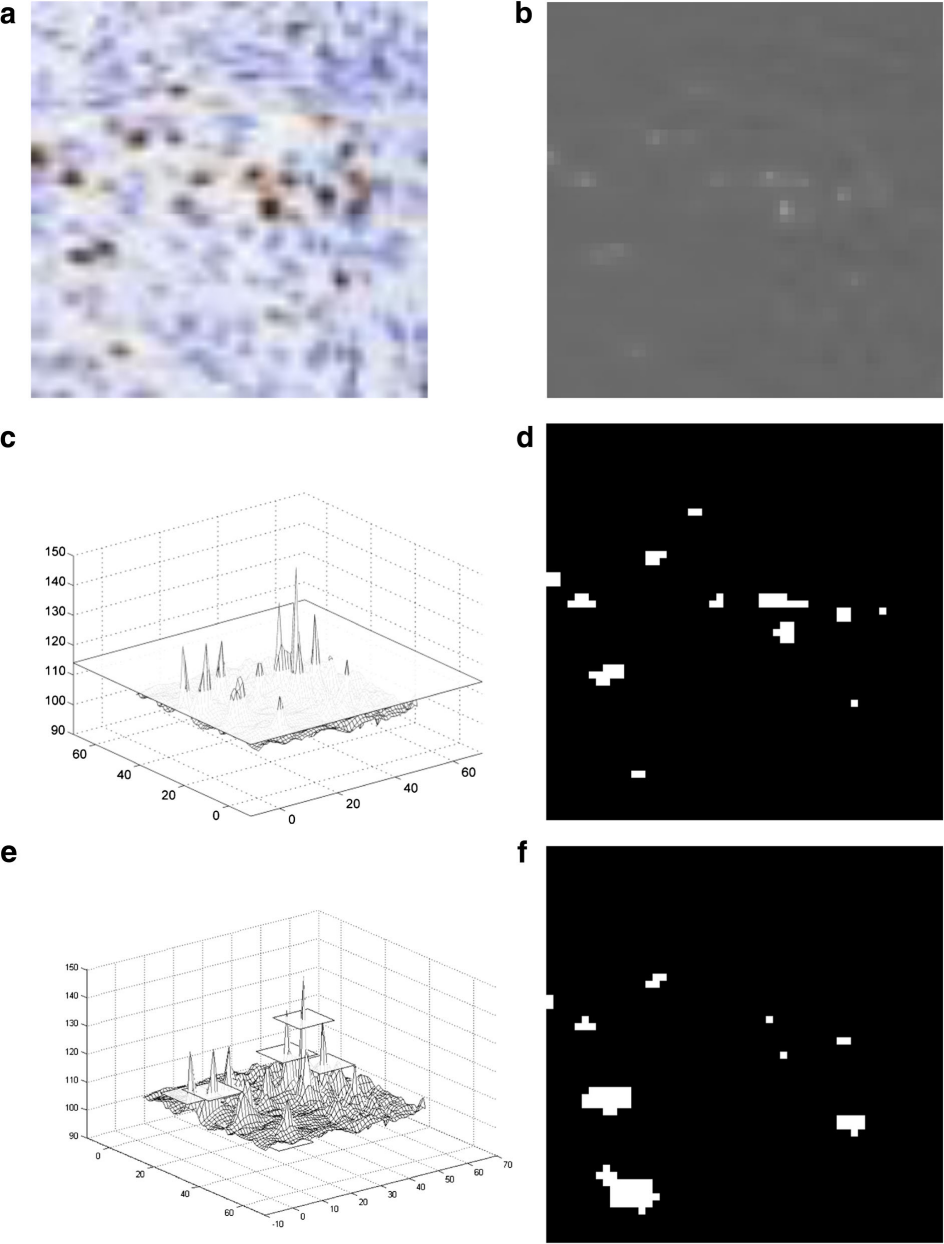
Example tumour region (**a**) with an altitude map of a utilized color channel (**b**) and cut-off plane for extended regional maxima (**c**) and minima (**e**) with the results of segmentations (**d** and **f**, respectively)

The natural approach to extended regional maxima is commonly selected to recognize objects, in our case immunopositive cells. However, area composed of a set of merged immunopositive cells, or selection of too low *h* value can lead to recognition of overestimated of object areas. The selection of higher *h* value effects omit of low immunoreactivity cells. Therefore, we applied the inverse of extended regional minima transformation, which leads to the expected results. In such a manner, the non-immunoreactive region of the tumour image (background and immunonegative cells) can be recognized as an extended regional minima with a cut-off of immunopositive cells. Thus, this approach was applied in our solution. In such a manner an immunopositive cell concentration map was obtained, after relocating each recognized object to its central point. Examples of tumour regions with an altitude map of utilized color channel (luminance from Luv color space) and cut-off plane for both approaches and results are presented in Fig. 7.

As can be observed, based on the regional minima transform most immunopositive cells can be segmented without overestimation of their areas. The number of recognized cells relates to the intensity of reactions in separate cells and can be controlled by the selection of *h* value. In contrast, the regional maxima transform returns some cells with overestimated areas and with the selection of the lower *h* value, and the separation of cells can be lost.

### Color artifact elimination

In the studied WSI a color artifact was sometimes visible in the processed FOV. Such an incident can significantly destabilize the image processing, especially color standardization, its transformation to other color spaces, and have an effect on morphological processing itself. We observed that a small color artifact can change the range of luminance representation of an FOV.

The proposed solution for artifact presence is to eliminate the portion of the lowest values of luminance in the FOV representation. We selected this percentage, 5 %, as a universal value in cases, where an artifact is not dominant in the image. This procedure is applied to each processing image. The detection of an individual artifact is performed by comparison of minimal values of luminance with its value after cut-off of 5 % of the lowest values.

### Selection of hot-spot fields - hot-spot gradual extinction algorithm

Hot-spots are located in the area with the highest concentration of immunopositive cell nuclei. An image of density distribution is used to detect the localization of these fields. The density map was created by counting the number of objects in each window (FOV) on the binary cell mask. In real cases, it is possible that the dominance proliferative Ki-67 index exists only in one area or in a few different areas. In all cases, selected hot-spot fields should represent diverse localizations with a high proliferation index. In order to prevent hot-spot selection only in particular area, we propose a solution called the “hot-spot gradual extinction algorithm,” which uses the penalty function first defined in paper [13]:

$$ penalty=1-\rho {\displaystyle \sum_i\frac{1}{{\left(\sqrt[]{{\left(x-{x}_i\right)}^2+{\left(y-{y}_i\right)}^2}\right)}^{0.5}}} $$

Based on this function, the value of each hot-spot candidate in (*x*,*y*) coordinates is reduced with a penalty factor. This factor relates to the number and distance from the selected hot-spot FOV (*x*_*i*_,*y*_*i*_). Thus, a region that is represented by a few selected FOVs can have its value decreased, so that other regions will be able to gain in value. This process is increased through hot-spot selection, so we term this the “gradual extinction” of regions.

As a result, factors such as localization and the proliferation index are necessary for correct hot-spot selection. The proposed solution with the penalty factor allows for hot-spot selection in diverse examples of specimens. If one area dominates, the proposed solution allows the selection of hot-spot fields from various localizations of the tumour.

Simultaneously, if other regions have significantly lower Ki-67 levels than the dominant region, the hot-spots will still be selected from the dominant region. Another matter that should be discussed is what specimen size should be taken into account in this process. It is possible and justified to take this factor into account during the penalty function and/or in terms of the number of fields selected fields for Ki-67 counting in a specimen. The following solutions can be proposed:

- If the area of the specimen is lower than the summarized area of 500 FOV, the number of selected hot-spot fields is restricted to (specimen_area/FOV_area)*20/500.
- In other cases, the standard procedure is applied
- If the specimen has a low compact area, the penalty factor can be reduced.

For the latter solution, the reference value of the specimen area and radius must be selected. The margin value is set to 100 FOV (area) and six radiuses of a circular field with an area equal to one standard FOV. If any of these candidates is not fulfilled, the penalty factor is reduced in the same proportion as the ratio of the effective area or radius to the reference value mentioned above.

In order to determine the Ki-67 index, each hot-spot field is quantitatively analyzed on full resolution images with the algorithm described in [33].

### Statistical analysis

With the aim of an evaluation of the concordance between the semi-automatic and automatic examination of Ki-67 proliferation index in WSI, the Spearman’s rho correlation coefficient and Bland-Altman plot were used. The comparisons were performed in each pair, e.g. between the experts’ results and between the automatic result and each of the expert’s results. Thus, we obtained the metric of examination agreement both generally and according to the Ki-67 index level, as well as identifying the mean displacement between expert and automatic results. Due to the lack of a uniform distribution of Ki-67 level and low cut-off values between the suggested WHO grades, Bland-Altman plots are also presented in the logarithmic scale for better result readability.

## Results

To establish one complete system for hot-spot detection, a lot of preliminary examinations of specific tasks were performed in this complex study and these have been presented in papers [12–14]. In this study, we would like compare the Ki-67 results obtained based on the automatic examination of specimens with the results of hot-spot fields selected by two experts. The database contains 104 cases of meningiomas: meningothelial 76 cases (73 %), atypical 22 cases (21 %), and anaplastic six cases (6 %). The two medical experts had extensive experience (noted as Expert A and Expert B) and were asked to select 20 hot-spot fields in each WSI, if they existed. It should be noted that this requirement is very hard to meet. Indeed, some specimens have a limited area and it is not possible to select that number of hot-spots. In contrast, manual control of field selection in a large specimen with a lot of hot-spot regions is difficult and practically impossible to maintain. Therefore, in practice, we obtained a different number of hot-spot fields. As a result, the 20 FOV (if such their number was indicated) with the maximal Ki-67 index were used for calculation.

In the automatic hot-spot selection process, 20 (if existing) FOV were obtained. Each was examined by an expert and every field with some technical problem (artifact, inappropriate stain, e.g. focal background staining, blurred, classification problem, etc.) was marked. The results were grouped according to the number of FOV with artifacts and a number of existing hot-spot FOV are presented in Table 1. It should be noted that sometimes an individual FOV with an artifact was also indicated by an expert in a set of hot-spot fields. However, a limited number of inappropriately selected FOV should not have a significant impact on the specimen examination.

**Table 1:** The results of Ki-67 evaluation grouped according to their range and no of hot-spot fields

			Ki-67 index			
System: no of artifacts	FOV per case	No of cases	System	Expert A	Expert B	Remarks
0	20	52	10.25 %	8.87 %	9.27 %	
	15–16	3	5.84 %	5.58 %	5.61 %	
1	20	16	8.35 %	6.02 %	6.62 %	Focal background staining, artifact
	8–15	3	4.80 %	3.24 %	3.67 %	Artifact
2	20	7	4.14 %	3.02 %	3.17 %	Focal background staining, blurred, alterations of the structure, e.g. coagulation
	8–13	3	5.72 %	4.89 %	6.91 %	Artifact
3	20	10	3.48 %	2.67 %	2.70 %	Artifact
	12–14	3	3.54 %	1.63 %	1.52 %	Artifact
2	20	1	17.19 %	6.12 %	5.83 %	Artifact
4	20	1	5.85 %	5.11 %	6.15 %	Stained vessels
5	20	2	4.58 %	3.51 %	4.72 %	Hemorrhages
6	20	1	6.01 %	4.47 %	4.86 %	Stained vessels
9	20	1	10.49 %	5.68 %	5.92 %	Focal background staining, alterations of the structure, e.g. coagulation, other artifacts
12	20	1	3.89 %	2.40 %	2.89 %	Stained vessels

When analyzing the results depicted in Table 1, it can be noticed that most of the cases (84) were automatically examined properly with two FOVs with a technical problem at the very most. The 13 others were selected with three problematic FOVs, including artifacts. Only seven specimens (depicted last in Table 1) required any comments. The first with two FOVs with artifacts has a part of its tissue with alterations of the structures, probably at the stage of the fixation process. The next with four and six inappropriate FOVs, and the last one, are specimens with an inappropriate staining in thin-wall vessels. This is the reason why our algorithm does not eliminate the vessels from the specimen map. Two specimens with five false hot-spot fields includes a lot of hemorrhages inside a tumour tissue which is difficult problem to area elimination as well as properly classification of the cells. The one case with nine selected inappropriate FOVs has a lot of technical problems, which cannot be detected automatically. Finally, these seven specimens should be excluded from the automatic quantitative analysis, due to their many technical problems in general.

**Fig. 8:**
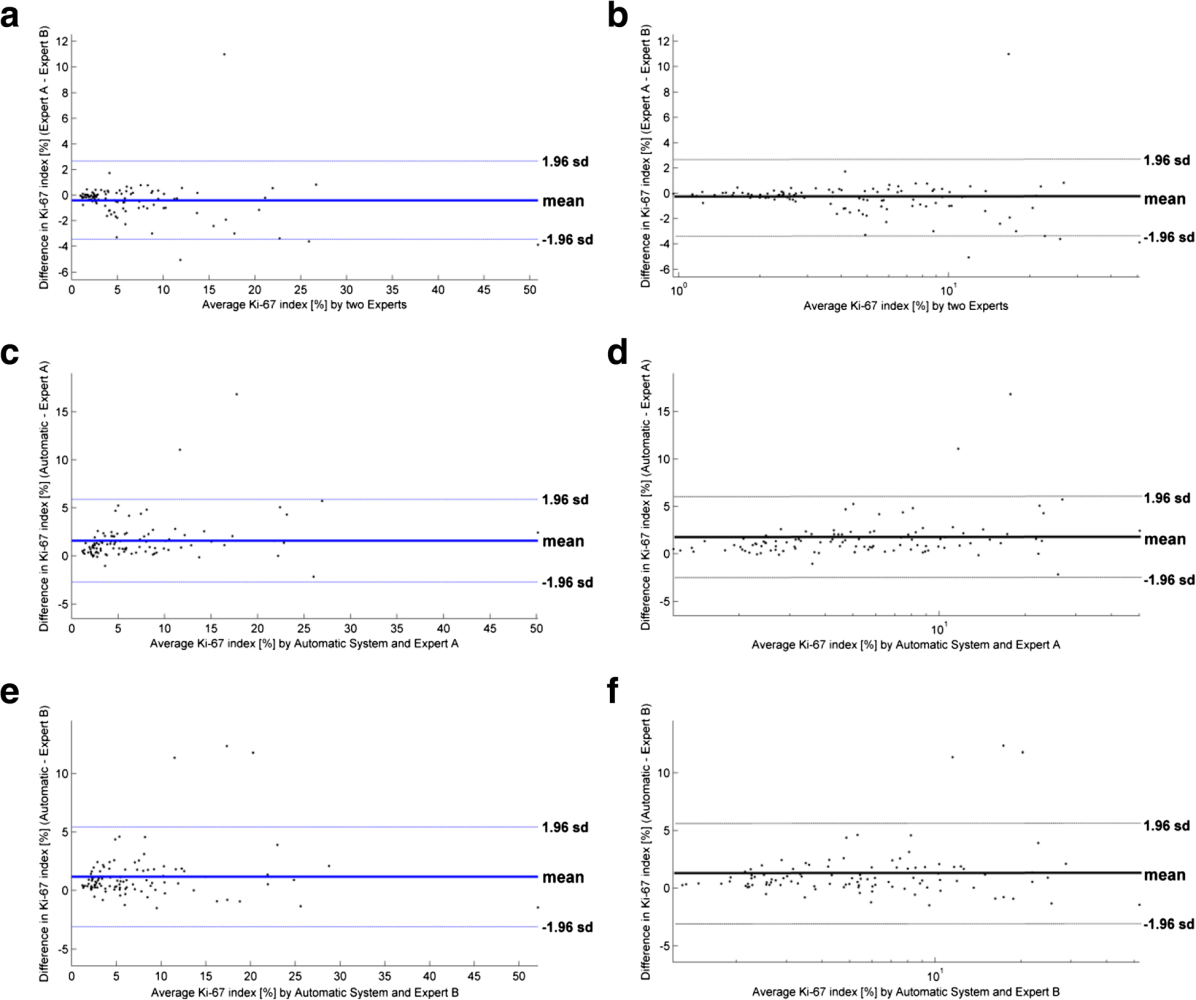
The Bland-Altman plots of differences between the two Experts (**a** and **b**), Automatic System and Expert A (**c** and **d**), and Automatic system and Expert B (**e** and **f**). The first plots are in a linear scale, whereas the second ones are in a logarithmic scale

The comparison of the results in relation to Ki-67 level was performed with the help of a Bland-Altman plot. The results are presented in Fig. 8 as a comparison between the experts’ results and between the automatic results and the results of each expert. We noticed that the difference of Ki-67 index estimation between the Experts (Fig. 8a and b) is on a low average level 0.41 %. This shows a slight overestimation of Ki-67 index by expert B in the upper range of values. Only one significant outlier is noticed. Comparison of the results of the Automatic System and Expert A shows that the average discrepancy equals 1.58 %, with the smallest value in the low level of Ki-67 index and the highest in the high level. In general, the Automatic system returned the highest value of Ki-67. Only two outliers are noticed in the high level of Ki-67 index. Very similar results were obtained in comparison with the annotations of the Automatic System and Expert B. The average discrepancy is 1.17 %, and three outliers can be noted. However, in this case some automatically established Ki-67 indexes are also lower than the ones obtained by Expert B.

To explain the differences in the Ki-67 estimations, the selected FOVs were compared and their localizations in WSI were studied. The authors observed that the FOVs selected by Expert A are more dispersed than the FOVs of Expert B. This fact is particularly revealed in high Ki-67 index level specimens, resulting in a higher Ki-67 index estimation by Expert B. Indeed, in such specimens Expert B selected a few more FOVs from the highest hot-spot regions, and as a result obtained a higher Ki-67 index value. In contrast, the higher FOV dispersion in Expert A and in the automatic examination of specimens leads to slightly lower Ki-67 values.

These results indicated that in general the Automatic System identifies better hot-spot areas, especially their maximum points. The scatter of differences increases with the Ki-67 level (see Bland-Altman plot in logarithmic scale in Fig. 8d and f). These phenomena form a complaint with data in quantitative examinations of histological specimens. For statistical confirmation of the consistency of results, the Spearman’s *rho* correlation coefficient was calculated. This is equal to 0.9774 between the results of both Experts, whereas between the automatic system and Expert A or B it is equal to 0.9590 or 0.9602, respectively (all *p* < 0.000005). All statistical examinations confirm the good level of agreement between the automatic quantitative evaluations of Ki-67 index and the experts’ results.

## Discussion

Computer methods and systems for histopathological evaluation of a specimen are a rapidly developing area. Digital imaging tools can be used as a support for medical experts. Image analysis improves the accuracy and reproducibility of pathologists’ interpretations, because interobserver and intraobserver variabilities are eliminated. As a result, this leads to providing better information for clinicians in treatment decisions for patients [34]. The application of automated image analysis tools provides a standard, reproducible, sensitive and specific method of biomarker quantitation, whilst the standard, manual procedure is based on manual scoring. The latter method is subjective, time-consuming, and characterized by intraobserver and interobserver variabilities [35]. Digital pathology tools have the potential to support diagnostic processes [36]. The example of application of automated image analysis algorithms for histopathological evaluation of a specimens will be available on MIAP web platform: *https://miap.wim.mil.pl* [37], starting late autumn 2016.

Different types of staining methods require dedicated algorithms for each image. The method for structure detection and evaluation of specimens are presented in the literature [5–10]. The methods for evaluation and analysis of tissue treated in a standard Hematoxylin & Eosin stain are presented in [7, 8]. This is a different staining method from Ki-67 staining, and the final images are different (have different colors). As a result, we cannot apply this solution in the analyzed cases. A distinct method is needed for evaluation of specimens stained with the Ki-67 staining method. The algorithms for cell segmentation and counting are presented in papers [5, 6]. However, the authors focused only on cell segmentation and counting problems. They did not propose a method of hot-spot area identification and selection, nor did they solve the artifact problem. H. Lu et al. proposed the application of the adaptive step finding technique for the automated selection of a hot-spot [11].

To the best of our knowledge, the problems of artifacts and spatial distribution of the selected hot-spot fields have not been solved in the literature. In this paper, we have presented a proposition for an algorithm to solve the hot-spot selection problem, the hot-spot distribution problem, the detection and counting of cells, and artifact detections. Solutions for specific tasks connected with hot-spot selection and artifact detection are presented in our previous studies [12–14]. In our study, we have detected artifacts, such as tissue folds, areas of hemorrhages/erythrocytes, and vessel walls. These areas are marked with similar colors to immunopositive cells, and can be classified wrongly. Their presence may interfere with the automatic examination of WSI. Specimen quality introduces limitations for the presented algorithm. This problem has not been solved in the present study. Different alterations of the structures in the tissue, blurring and dye stains are hard to detect in WSI. The detection and elimination of small vessel walls is limited, because the increasing of vessel wall detection sensitivity causes wrong classification of the immunopositive cells, in cases of a high Ki-67 index in the specimen. The advantages of the presented algorithm are: a solution to the spatial distribution of the hot-spot problem; elimination of artifacts such as folds, hemorrhages/erythrocytes, and vessel walls; reproducibility of results; and short calculation time.

Recently, content image analysis oriented to WSI has become a focus of interest for many researchers [38]. The identification of tissue pathology, based on textural analysis in different image resolutions and staining, has been developed with significant successes. The connection between this idea and our method will give a new potential in digital diagnostic pathology.

## Conclusion

In this paper, the authors proposed a complete system for automatic hot-spot selection and Ki-67 examination in specimens of meningiomas. The proposed algorithm, based on textural descriptors, classifiers and mathematical morphology, is able to detect the specimen map and identify artifacts such as hemorrhages (vs. erythrocytes both intravascular or extravasated), tissue folds, and stained vessel walls. The proposed strategy of hot-spot field selection, called the hot-spot gradual extinction algorithm, provides an efficient tool for simulation of human examination. The presented results confirm that the proposed automatic selection of hot-spot fields and examination of Ki-67 in meningiomas can be introduced in pathomorphological practice in the near future.

## Additional file

Additional file 1: The results of the manual and automatic Ki-67 index evaluation for each of cases. (XLSX 16 kb)

## Abbreviations

CORF: Combination of receptive fields
FOV: Field of view
LBP: Local binary patterns
SVM: Support vector machine
WSI: Whole slide image
